# Quality of life and socio-demographic factors associated with nutritional risk in Brazilian community-dwelling individuals aged 80 and over: cluster analysis and ensemble methods

**DOI:** 10.3389/fnut.2023.1183058

**Published:** 2024-01-03

**Authors:** Guilherme Carlos Brech, Vanderlei Carneiro da Silva, Angelica Castilho Alonso, Adriana Machado-Lima, Daiane Fuga da Silva, Glaucia Pegorari Micillo, Marta Ferreira Bastos, Rita de Cassia de Aquino

**Affiliations:** ^1^Postgraduate Program in Aging Sciences, Universidade São Judas Tadeu, São Paulo, Brazil; ^2^Laboratory for the Study of Movement, Department of Orthopedics and Traumatology, School of Medicine, Universidade de São Paulo, São Paulo, Brazil

**Keywords:** aged, quality of life, nutritional risk, cross-sectional study, machine learning

## Abstract

**Introduction:**

The aim of the present study was to use cluster analysis and ensemble methods to evaluate the association between quality of life, socio-demographic factors to predict nutritional risk in community-dwelling Brazilians aged 80 and over.

**Methods:**

This cross-sectional study included 104 individuals, both sexes, from different community locations. Firstly, the participants answered the sociodemographic questionnaire, and were sampled for anthropometric data. Subsequently, the Mini-Mental State Examination (MMSE) was applied, and Mini Nutritional Assessment Questionnaire (MAN) was used to evaluate their nutritional status. Finally, quality of life (QoL) was assessed by a brief version of World Health Organizations’ Quality of Life (WHOQOL-BREF) questionnaire and its older adults’ version (WHOQOL-OLD).

**Results:**

The K-means algorithm was used to identify clusters of individuals regarding quality-of-life characteristics. In addition, Random Forest (RF) and eXtreme Gradient Boosting (XGBoost) algorithms were used to predict nutritional risk. Four major clusters were derived. Although there was a higher proportion of individuals aged 80 and over with nutritional risk in cluster 2 and a lower proportion in cluster 3, there was no statistically significant association. Cluster 1 showed the highest scores for psychological, social, and environmental domains, while cluster 4 exhibited the worst scores for the social and environmental domains of WHOQOL-BREF and for autonomy, past, present, and future activities, and intimacy of WHOQOL-OLD.

**Conclusion:**

Handgrip, household income, and MMSE were the most important predictors of nutritional. On the other hand, sex, self-reported health, and number of teeth showed the lowest levels of influence in the construction of models to evaluate nutritional risk. Taken together, there was no association between clusters based on quality-of-life domains and nutritional risk, however, predictive models can be used as a complementary tool to evaluate nutritional risk in individuals aged 80 and over.

## 1 Introduction

The over 80 years demographic group has been increasing steadily worldwide and, although this fact should be celebrated by humanity, increased life expectancy may bring along a higher frequency of chronic diseases, which could impair a person’s ability to perform daily activities, as well as their independence and autonomy. Consequently, it may enhance physical fragilities, as well as social and psychological vulnerabilities, which could trigger negative feelings and decrease aging-associated quality of life (1).

Quality of life (QoL) has been defined by the World Health Organization (WHO) as the subjects’ perception about socio-cultural and value systems in which they are inserted and regarding their goals, expectations, and concerns. Therefore, to keep the general population’s QoL at a higher level, it is necessary to consider some aspects, namely physical and psychological health, as well as to obtain satisfactory levels in other domains, such as psychological, social relations, and environmental (2). In addition, higher scores of QoL for older adults considers additional domains, such as autonomy, sensorial functioning, social participation, past, present, and future activities, as well as death and dying.

In Brazil, a country in development, individuals aged 80 and over represent 2% of the population (3); with an estimated increase of this group to 2.2% by 2030, and 4.07% by 2040 (4). Thus, this accelerated growing of the number of oldest-old in a country with countless socioeconomic inequalities must be considered in an attempt to improve public policies focusing on lifelong health.

Dietary and lifestyle patterns are undeniably relevant for both the physical and psychological conditions of all individuals, contributing for prevention of health-related problems as well as maintenance, and promotion of health; they also contribute to the well-being that impacts quality of life (5). However, the Brazilian Institute of Geography and Statistics has reported that food insecurity in the Brazilian population has increased from 2013 to 2018 (6). Salles-Costa et al. (7) described an increased number of people experiencing food insecurity in the aforementioned period (from 2.9 to 5.6 million). On the other hand, households having at least one older adult present lower prevalence of food insecurity, probably due to the Continuous Benefit Installment program, which also contributed for poverty reduction in Brazil from 2004 to 2013 (8).

Food insecurity (FI) is characterized by irregular access to food, and has a negative impact on food intake, nutritional status and health of older people. Approximately 2.37 billion people worldwide were affected by moderate or severe food insecurity in 2021 due to environmental and socio-economic conditions (9). A recent systematic review included 22 studies of different worldwide regions, being 18.2% Europeans 13.6% Asian, 9.1% African, and 13.6% Latin American populations, and concluded food insecurity was associated with malnutrition (45.5%) and with overweight (27.3%) in older adults (10). In Portugal (11) and Iceland (12) nutritional risk was considered a critical factor for the health of older adults’, primarily affecting those living alone, which increases the health concerns as well as QoL of older subjects (11, 12).

The prevalence of FI in studies among Brazilian community-dwelling older people varies, depending on the region. In 2021, 7% of the population were living in poverty and 19% in extreme poverty (<US$1.90 *per capita*/day), revealing the social inequalities of Brazil. In a recent study on food insecurity in older people covered by a program of the Family Health Strategy in Northeast Brazil, the prevalence was 63.5% in households with older people (38.4% mild FI and 25.1% moderate/severe FI), a scenario that was exacerbated after the pandemic period (9).

Although there are studies about older adults, only a handful of them have associated quality of life and socio-demographic factors with nutritional risk, which renders studies using machine learning of fundamental importance, particularly for a country with so many inequalities and where longevity increases at such a high pace. The aim of the present study was to use cluster analysis and ensemble methods to evaluate the association between quality of life, socio-demographic factors to predict nutritional risk in community-dwelling Brazilians aged 80 and over.

## 2 Materials and methods

### 2.1 Study design and ethics

This cross-sectional study was approved by the Research Ethics Committee of São Judas Tadeu University (CAAE 49987615.3.0000.5404 and approval number 09069419.8.0000.0089).

### 2.2 Participants

Participants were invited to participate from partner reference centers of the state of São Paulo, Brazil, where ongoing health promotion activities are performed for older adults. The invitation was writing and by word of mouth. Thus, this was a non-probability convenience sample, in which they were included aged 80 and over subjects, able to communicate and cognition preserved enough to answer the questionnaire inclusion criteria. An investigator carefully presented the aims, risks, benefits of the study, and freedom of participation, as well as confidentiality for all aged 80 and over subjects, moment who had opportunity to decline. Then, who agreed to participate of the study signed an information sheet and a consent form. They were excluded who did not finish answering the questionnaires too were.

Thus, the data collection occurred from October 2016 to October 2017, in four Institutions from São Paulo state: Casa do Idoso Norte (North Older Adults’ House, São José dos Campos city), Centro Integrado de Saúde e Educação (Moacyr Rodrigues Moacyr Rodrigues- Aging Health and Education Integrated Center, São Caetano do Sul city), Escola Superior de Educação Física (Superior School of Physical Education, Jundiaí city) and Universidade São Judas Tadeu (São Paulo city). The institutions located in São José dos Campos, São Caetano do Sul and Jundiaí are reference centers for the older adults’ population, since they have medical care and socializing spaces exclusive for people over 60.

The self-reported sociodemographic characteristics (age, sex, ethno-racial features, marital status, schooling, household income), self-reported health conditions and number of teeth were obtained from aged 80 and over subjects with the printed questionnaire by interview with a multidisciplinary staff previously trained and with experience in gerontological assessment (biomedical, physiotherapist, psychologists, and nutritionist).

### 2.3 Mini-mental state examination

The Mini-Mental State Examination (MMSE) (13) was used to assess the participants’ cognitive status. MMSE comprises questions divided into seven categories: orientation regarding time and place (5 points each), memorizing of three words (3 points), attention and simple mathematical calculation (5 points), remembering of three words (3 points), language (aphasia and apraxia, 8 points) and constructional skills (1 point). The MMSE score ranged from a minimum of 0 to a maximum total of 30 points. The cut-off point for cognitive decline considers schooling level, and this study used 18–19 and 24–25, according to the absence and presence of previous formal schooling, respectively (14).

### 2.4 Quality of life

QoL of the individuals aged 80 and over was evaluated using the WHOQOL-BREF and WHOQOL-OLD instruments. The WHOQOL-BREF was translated and validated in Brazil by Fleck et al. (15), and it consists of 26 items, two of which relate to global QoL and health in general, and the remaining 24 are organized into four domains (physical, psychological, environmental, and social relationships). The WHOQOL-OLD is a specific instrument for evaluation of QoL in older adults, and it was validated for Brazil’s older population by Chachamovich et al. (16). This instrument comprises 24 items divided into six domains: sensory abilities, autonomy, past-present-future activities, social participation, death and dying, and intimacy; it should be applied along with WHOQOL-BREF. In both instruments, the scoring answer is based on the Likert scale, varying from 1 to 5, according to satisfaction degree. The final scores vary from 0 to 100, and they are proportional with QoL; a score of 100 means the best QoL.

### 2.5 Strength test

Maximal handgrip strength was determined using a hydraulic hand dynamometer (model Jamar® by JLW Instruments, Chicago, IL, United States). All participants were tested while seated, elbow flexed at 90° with forearm and wrist in a neutral position, hips and knees flexed at 90°, feet on the ground, in accordance with the guidelines of the American Society of Hand Therapists (17). The testing protocol consisted of three repetitions of 5 s in maximal isometric contractions of the dominant hand, with a rest period of at least 60 s. The highest strength value among the three attempts was considered for analysis, and results were shown in kilogram/force (Kg/f).

### 2.6 Mini nutritional assessment^®^ and short form

The MNA® is a validated nutrition screening tool to identify individuals aged 65 and over at nutritional risk (18). Originally, the instrument comprised 18 questions, and the MNA Short Form (19) consists of 6 questions and streamlines the screening process while retaining the validity and accuracy of the original MNA® to identify older adults who are malnourished or at risk of malnutrition. The MNA-SF comprises simple measurements and six questions that can be completed in less than 5 min: anthropometric measurements (body mass index, weight loss); global assessment (mobility); and dietary questionnaire and subjective assessment (food intake, neuropsychological problems, acute disease). A total score of MNA-SF <8, 8–11, and > 11 indicates malnutrition, risk of malnutrition, and no malnutrition, respectively. Those who did not complete the questionnaire were excluded.

### 2.7 Statistical analysis

All analyzes were performed in R, version 4.1.0 (R Foundation for Statistical Computing). Data were tested for normality using the Shapiro–Wilk test, revealing non-normal distribution. Kruskal-Wallis, Bonferroni, and Dunn’s nonparametric tests were used to evaluate the presence of differences in sociodemographic (age, sex, race, marital status, schooling, household income) and health variables (self-reported health and number of teeth) by cluster, in muscular strength, MMSE, WHOQOL-BREF and WHOQOL-OLD domains. The numerical variables were presented as medians and interquartile ranges, or mean and standard deviation, while categorical variables were shown as frequencies (%). Associations between the categorical variables were tested using Chi-squared and Fisher Exact tests. The level of significance was established at 5% (*p* ≤ 0.05) for all analyzes.

**Figure 1:**
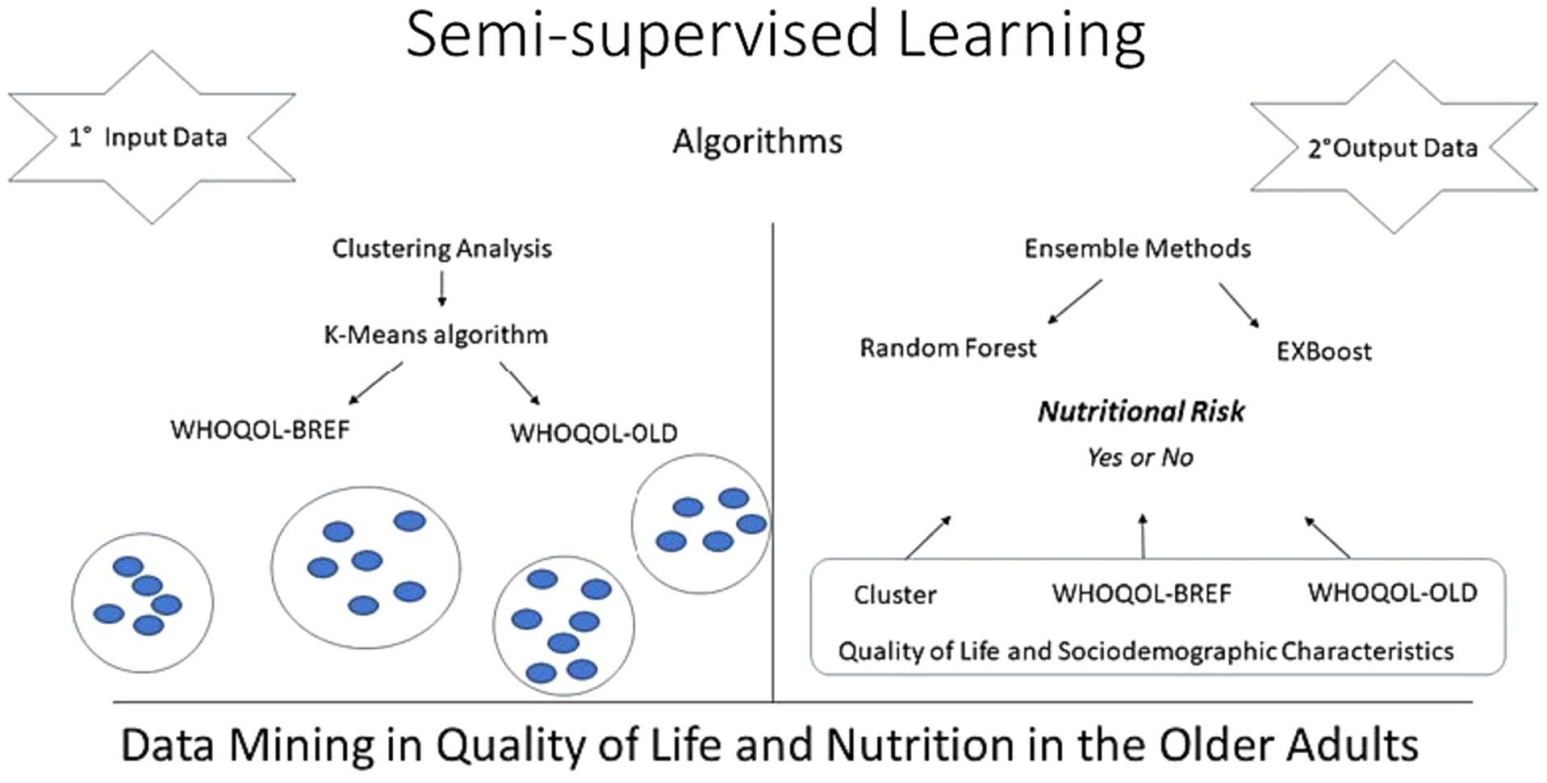
Conceptual model of quality of life and nutritional risk in the older adults.

Initially, clustering analysis (unsupervised machine learning) was used to derive groups of participants based on quality-of-life scores, and the association of the clusters with nutritional risk was tested. Subsequently, two classifier algorithms were used, Random Forest and eXtreme Gradient Boosting based on the ensemble strategy as part of the techniques of supervised machine learning to predict nutritional risk (Figure 1).

The characteristics of each machine learning algorithm is briefly described below.

#### 2.7.1 Machine learning

One of the most useful tasks in ML is that of labeling, assigning each data point a certain tag, label, or a numerical value according to some prespecified criterion. Semi-supervised or partially supervised learning refers to a class of ML techniques which combine labeled and unlabeled data to build classifiers (20). Additionally, an ensemble method is a set of classifiers whose individual decisions are combined in some way (typically by weighted or unweighted voting) to classify new examples. Ensemble methods are known to perform better than other algorithms, because they help to reduce false positive rates (21). Random Forest and eXtreme Gradient Boosting are based on the ensemble strategy and perform well in many tasks.

#### 2.7.2 Unsupervised learning and clustering analysis

**Figure 2:**
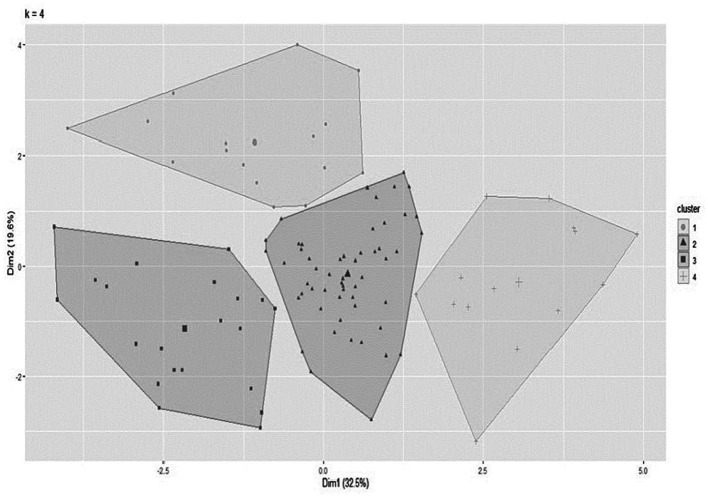
Cluster of individuals aged 80 and over performed by K-Means algorithm.

K-means clustering algorithm was used to identify the clusters based on WHOQOL-BREF and WHOQOL-OLD domains. The following packages were used: cluster and factoextra. The QoL scores were converted to z-scores and input into the algorithm. Standardized data were used to ensure all features have equal influence on the clustering procedure. Clustering distance measurements were carried out using Euclidean distances. Each data point was categorized by calculating the distance between the point to each group centroid and then classifying the point closest to it (22). Centroids were recomputed based on the classified points, and the process was then repeated until the centroids no longer changed. Four clusters were retained considering homogeneity in the groups that were derived in the analysis (Figure 2) and considering the optimal number of clusters by average silhouette width (Supplementary Figure S1). Interpretability of characteristics for each group was examined to confirm the optimal number of clusters, and whether a group was sufficiently large for an adequate statistical power, that is, at least 10% of the total sample (23).

#### 2.7.3 Supervised learning, random forest, and XGBoost algorithm

The Random Forest (RF) and eXtreme Gradient Boosting (XGBoost) were used to predict the nutritional risk of each participant. The training stage of the classifier algorithms was based on the following predictors: age, sex, household income, self-reported health, handgrip, MMSE, number of teeth. These predictors were defined considering the models’ best performance in the test stage and evaluation metrics, such as accuracy and area under the curve. Sociodemographic data were used together with cluster and QoL characteristics to build three different models: (1) based on the Cluster; (2) WHOQOL-BREF; (3) WHOQOL-OLD.

The RF algorithm generates several decision trees, and each tree is trained with a random distribution. A major advantage of the RF is the ease of measuring the relative importance of each attribute for the prediction by analyzing how many nodes in the trees use a given attribute to reduce the overall impurity of the forest (22). The RF model was built through the Random Forest function, which is part of the package of the same name. Analysis was performed using the following established basic parameters (Supplementary Table S1): number of trees, minimum size of terminal nodes, number of variables used in each tree, and seed for reproducibility. The eXtreme Gradient Boosting algorithm (XGBoost) is also used as an efficient, popular implementation of gradient-boosted decision trees. XGBoost strictly prioritizes computational speed and model performance, with good accuracy using most data sets. The analysis was performed using the following established, basic parameters (Supplementary Table S1): number of trees, minimum size of terminal nodes and number of variables used in each tree, max number of boosting iterations, maximum depth of a tree, and seed for reproducibility.

#### 2.7.4 Feature selection and model assessment

The predictors used by the classifier algorithms were ranked according to the importance of their influence on the class prediction (with or without nutritional risk). The order of importance was based on the importance function of a RF model (randomForest package) and XGBoost model (EIX package). Concordance between the outcome class (nutritional risk) and the classification scheme provided by the algorithms was calculated. The accuracy was determined using confusionMatrix (e1071 package); in new individuals, for whom the risk label was not known by the algorithm, only information regarding the sociodemographic data and QoL characteristics were used. In general, values equal to 0.5 correspond to the performance of a random classifier, values smaller than 0.6 (and greater than 0.5) indicate moderate predictive performance and values greater than 0.7 indicate good predictive performance. Furthermore, other evaluation metrics were calculated, such as sensitivity, specificity, area under curve, positive and negative predictive values (24).

## 3 Results

The present study managed to recruit a sample of 104 older people in the period determined for collection, mostly women (69.23%), who declared themselves white (82.69%), with a median age of 82 years and 4 years of age education (Table 1).

**Table 1:** Description of sociodemographic and nutritional status characteristics by cluster.

	Overall		Cluster 1		Cluster 2		Cluster 3		Cluster 4		*p* value
	*n*	%	*n*	%	*n*	%	*n*	%	*n*	%	
Nutritional Status*											
Without risk	72	70.59	11	68.75	12	57.14	36	70.59	9	64.29	0.717
With risk	30	29.41	5	31.25	9	42.86	15	29.41	5	35.71	
Sex											
Male	32	30.77	4	25.00	6	28.57	12	22.64	10	71.43	<0.050
Female	72	69.23	12	75.00	15	71.43	41	77.36	4	28.57	
Ethno-racial features											
Not White	18	17.31	2	12.50	4	19.05	9	16.98	3	21.43	0.922
White	86	82.69	14	87.50	17	80.95	44	83.02	11	78.57	
Married											
No	67	64.42	10	62.50	15	71.43	38	71.70	4	28.57	<0.050
Yes	37	35.58	6	37.50	6	28.57	15	28.30	10	71.43	
Self-report health*											
Worst	24	23.53	2	12.50	4	19.05	14	26.92	4	30.77	0.632
Equal	61	59.80	11	68.75	12	57.14	32	61.54	6	46.15	
Better	17	16.67	3	18.75	5	23.81	6	11.54	3	23.08	

	Median	IQR	Median	IQR	Median	IQR	Median	IQR	Median	IQR	
Age (years)	82	80–84	81	80–84	81	80–84	82	80–84	83	81–88	
Schooling (years)	4	4–8	6	4–11	4	4–8	4	4–8	4	4–8	
Household Income	1874	0–3,368	1887	0–4,168	2000	0–3,300	1874	0–3,000	1,068	0–3,500	
Hand Grip	18	14–23	22	16–25	20	15–22	17	12–22	18	16–23	
Mini Mental	25	22–27	25	21–26	25	22–26	26	21–28	23	22–26	
Number of Teeth	0	0–1	0	0–2	0	0–1	0	0–1	0	0–1	

*P* values are derived from Chi-Squared or Fisher Exact tests (continuous and categorical variables). The level of significance was established at 5% (*p* ≤ 0.050). IQR, interquartile range. *Exclusion of 2 individuals aged 80 and over presenting malnutrition (*n* = 102).

Figure 2 illustrates the result of the final cluster analysis using the K-means algorithm, resulting in four groups used to perform clustering. Figure 2 shows the clusters that were derived based on the QoL data. The sum between the two dimensions, which corresponds to the Y (19.6%) and X (32.5%) axes of the graph, explains a total of 52.1% of the QoL variability in the sample. Furthermore, it shows that there was no overlap between the clusters, a fact that indicates that the K-means algorithm was able to group individuals in such a way that participants from the same group are more homogeneous among themselves when compared to participants who are in other clusters. The figure is used as an initial indicator of model performance, additionally comparisons between clusters are described in Tables 1, 2, which allow us to characterize each cluster and make comparisons between them. Other subdivisions were tested during the analysis, but they did not show better intra-cluster homogeneity and differences between the groups, resulting in overlapping and small subgroups (data not shown).

**Table 2:** Description of scores of all domains of quality of life by cluster.

	Cluster 1		Cluster 2		Cluster 3		Cluster 4	
	Mean	SD	Mean	SD	Mean	SD	Mean	SD
WHOQOL-BREF								
DOM1–Physical	81.25^b,c^	17.37	74.58^e^	15.41	66.46^b^	13.23	59.69^c,e^	10.98
DOM2–Psychological	86.20^a,b,c^	7.09	75.98^a e^	9.94	69.77^b,f^	9.05	58.63^c,f^	8.56
DOM3–Social	90.10^a,b,c^	8.18	71.57^a,e^	13.20	68.26^b,f^	12.96	57.74^c,e,f^	10.06
DOM4–Environmental	87.89^a,b,c^	8.68	73.16^a,e^	9.25	67.89^b,f^	9.35	55.80^c,e,f^	6.57
WHOQOL-OLD								
Autonomy	58.33^a,c^	13.93	87.50^a,d,e^	10.27	63.35^d,f^	11.40	44.7^c,e,f^	12.46
Past, Present and Future Activities	71.88^a,c^	16.32	90.77^a,d,e^	8.97	73.58^d,f^	11.27	55.29^c,e,f^	17.28
Social Participation	71.35^a^	14.46	90.48^a,d,e^	9.81	69.32^d^	12.19	60.10^e^	13.63
Death and Dying	62.50	25.56	80.95	20.10	63.49	27.42	67.79	26.98
Sensory Abilities*	62.65	43.75–93.75	87.50^e^	81.25–93.75	75.00	62.50–93.75	50.00^e^	37.50–81.25
Intimacy*	68.75^a,b,c^	59.38–81.25	93.75^a,d,e^	87.50–100.00	75.00^b,d,f^	68.75–81.25	50.00^c,e,f^	50.00–56.25

*P* values were derived from a Bonferroni test or Dunn’s test, and statistical significance were represented with different letters: ^a^cluster 1 e 2; ^b^cluster 1 e 3; ^c^cluster 1 e 4; ^d^cluster 2 e 3; ^e^cluster 2 e 4; ^f^cluster 3 e 4. *Median and Interquartile Range. DOM, domain.

The clusters’ sociodemographic characteristics are shown in Table 1. From the original data set (*n* = 104), four major clusters were derived: cluster 1 (*n* = 16, 15.7%), cluster 2 (*n* = 21, 20.6%), cluster 3 (*n* = 51, 50.0%) and cluster 4 (*n* = 14, 13.7%). Although there was a higher proportion of individuals aged 80 and over with nutritional risk in cluster 2, and a smaller proportion in cluster 3, there was no statistical association between nutritional risk and the cluster derived from QoL data (WHOQOL-BREF and WHOQOL-OLD scores). On the other hand, there were more men in cluster 4, while cluster 3 had the highest proportion of women (*p* < 0.05). In addition, there was a higher proportion of individuals who were unmarried in cluster 3 (*p* < 0.05).

Table 2 shows the mean/median and standard deviation/interquartile range for WHOQOL-BREF and OLD scores by cluster of individuals aged 80 and over. Regarding WHOQOL-BREF, higher scores were present in cluster 1 and 2 when compared to clusters 3 and 4 for the physical domain (*p* < 0.05). Also, cluster 1 showed the highest scores for psychological, social, and environmental domains, while cluster 4 presented the worst scores for social and environmental domains. Similar findings were found regarding cluster 4 in the WHOQOL-OLD analysis, the worst scores in comparison to other clusters were for the following domains: autonomy, past-present-future activities, and intimacy. On the other hand, the highest scores for the abovementioned domains were observed in cluster 2, which was also the case for the social participation domain. No statistical difference between clusters was noteworthy for the death and dying domain (*p* > 0.05).

Regardless of the classifier built with QoL data according to WHOQOL-BREF, OLD or the Cluster, the RF algorithm showed the highest values for accuracy and area under the curve. Although the XGBoost algorithm exhibited greater accuracy (>0.70) for all models based on predictors of quality of life associated to sociodemographic characteristics, its specificity was the lowest when compared to the RF algorithm. This finding suggests a lower ability of the XGBoost algorithm to identify individuals at nutritional risk. All metrics used to evaluate the performance of the RF and XGBoost models are shown in Table 3.

**Table 3:** Performance measures of the ensemble algorithms.

Algorithm	Random forest			XGBoost		
Random forest	Cluster	BREF	OLD	Cluster	BREF	OLD
Accuracy	0.80	0.78	0.87	0.74	0.71	0.76
95% CI	0.65; 0.90	0.63; 0.89	0.73; 0.95	0.66; 0.84	0.56; 0.80	0.55; 0.83
Sensibility	0.80	0.80	0.85	0.80	0.75	0.85
Specificity	0.80	0.76	0.88	0.64	0.68	0.60
Positive predictive value	0.76	0.73	0.85	0.64	0.65	0.63
Negative predictive value	0.83	0.83	0.88	0.80	0.77	0.83
Prevalence	0.44	0.44	0.44	0.44	0.44	0.44
Detection Rate	0.36	0.36	0.38	0.35	0.33	0.38
AUC	0.87	0.86	0.85	0.72	0.72	0.73

Predictors: age, sex, income, health assessment, hand grip, mini mental, number of teeth. AUC, area under the curve measure.

**Figure 3:**
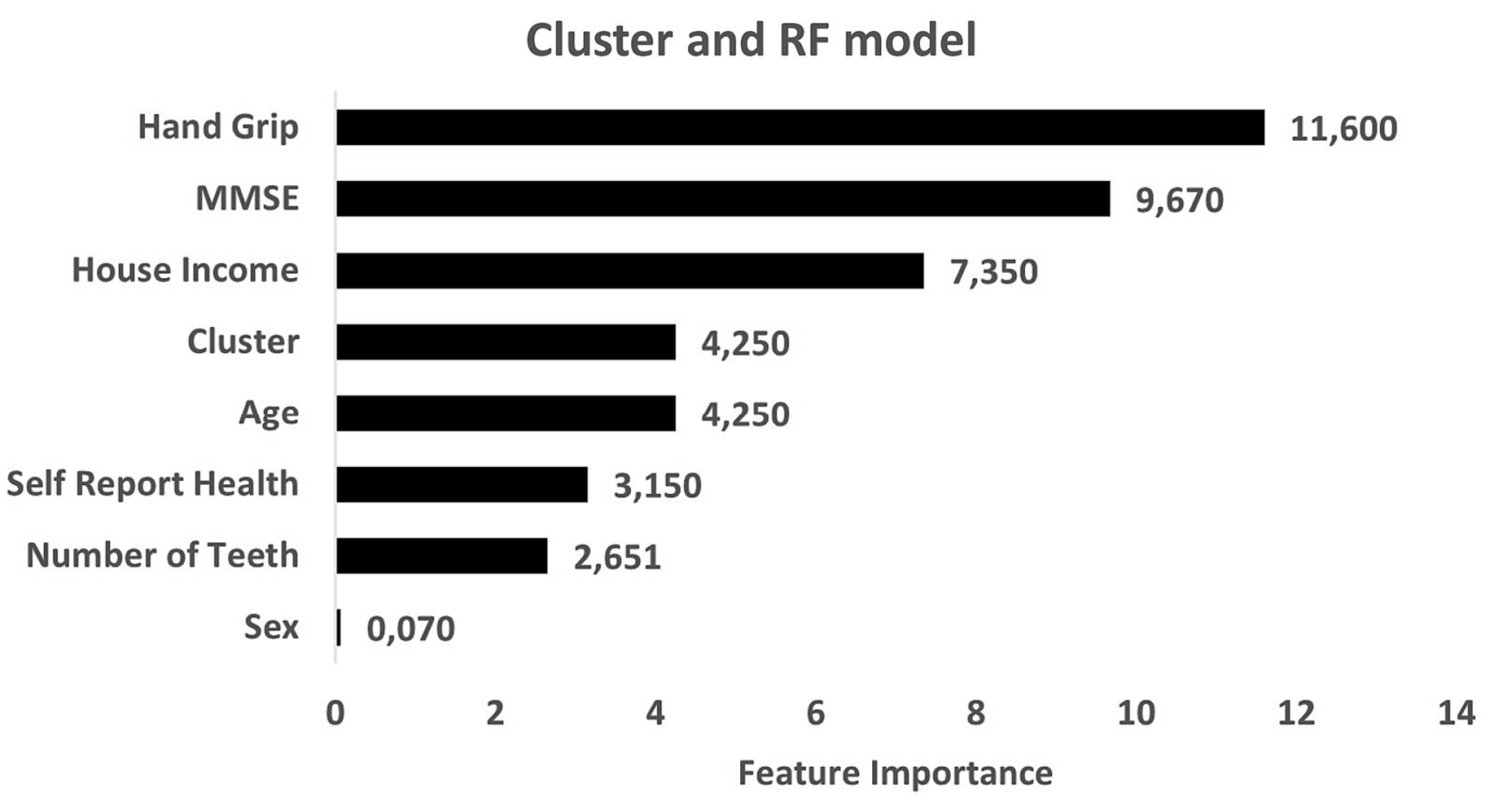
Predictors of nutritional risk with cluster quality of life and sociodemographic characteristics, self-reported-health and number of teeth, Mini-Mental State Examination (MMSE), and handgrip to individuals aged 80 and over analyzed by Random Forest model.

**Figure 4:**
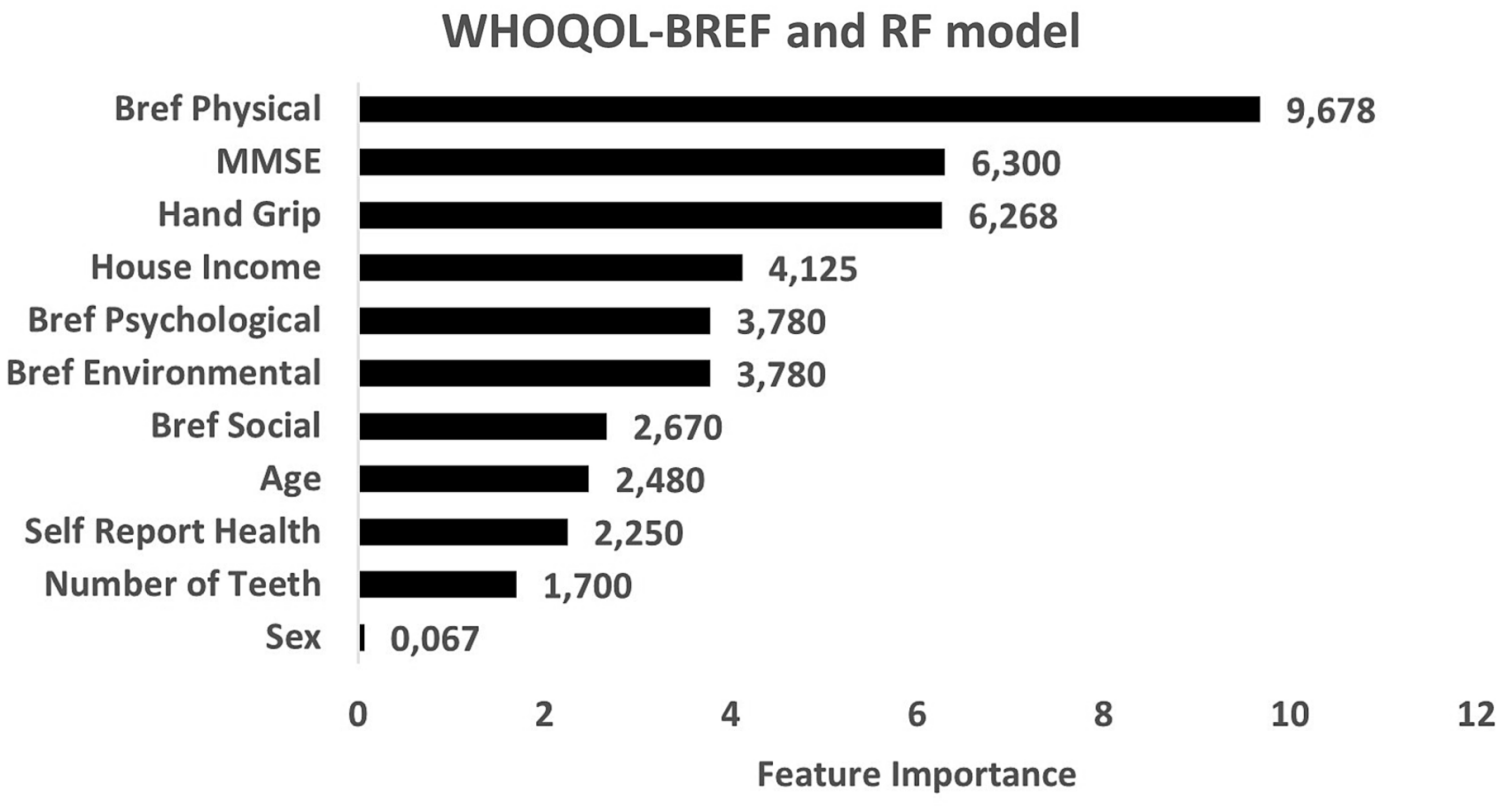
Predictors of nutritional risk with scores of WHOQOL-BREF and sociodemographic characteristics, self-reported-health and number of teeth, Mini-Mental State Examination (MMSE), and handgrip to individuals aged 80 and over analyzed by Random Forest model.

**Figure 5:**
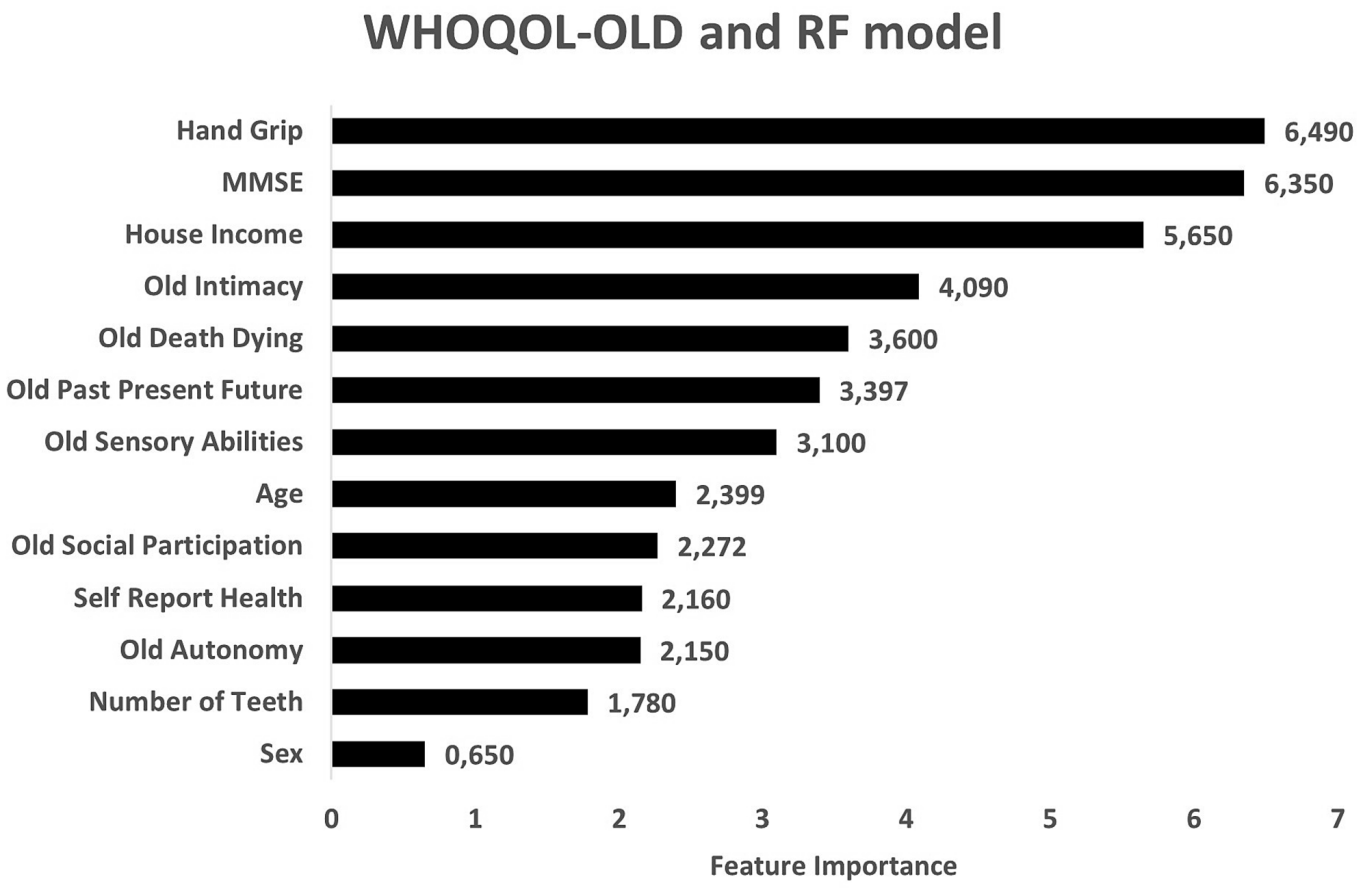
Predictors of nutritional risk with scores of WHOQOL-OLD and sociodemographic characteristics, self-reported-health and number of teeth, Mini-Mental State Examination (MMSE), and handgrip to individuals aged 80 and over analyzed by Random Forest model.

Regarding level of importance, handgrip, household income, and MMSE were the most important predictors of nutritional risks. On the other hand, sex, number of teeth and self-reported health showed the lowest levels of influence in the construction of models to evaluate nutritional risk (Figure 3). RF models exhibited better consistency to identify physical domain scores in the WHOQOL-BREF instrument (Figure 4). Feature selection was performed for both algorithms, and the results of the XGBoost model are presented as Supplementary material, due its lower performance, while the main predictors of nutritional risk analyzed by RF were ordered in accordance with their level of importance in Figures 3–5.

## 4 Discussion

The findings of the current study showed that, among the four clusters formed by the K-means algorithm, the predictors of nutritional risk for Brazilian community-dwelling individuals aged 80 and over were lower muscular strength, income, and cognitive capacity. Although there was no difference between clusters in relation nutritional risk, there was an association with female sex and unmarried status. Regarding quality of life, there was no association between nutritional risk and the clusters derived from the analysis. In the ensemble methods, the RF analysis had the best accuracy, sensitivity, and specificity, and the predictors with the highest importance in the model constructions were handgrip, household income, and MMSE.

Overall, the main predictors of nutritional risk for older adults were those associated with frailty, which, in the present study, were assessed through physical condition and cognition. According to Morley et al. (25), frailty is characterized as a dynamic and multifactorial clinical state that determines imbalance of homeostatic reserves associated with a reduction in the ability to respond to minor injuries, and an increased risk of dependence; quality of life is directly impacted by increased dependence. The systematic review and meta-analysis conducted by Kojima et al. (26) demonstrated a consistent inverse association between frailty/pre-frailty and quality of life among community-dwelling older adults, despite high heterogeneity and possible risk of biases in this population.

Furthermore, the present study identified that the intrinsic capacity of individuals aged 80 and over was a predictor of nutritional risk, represented by handgrip and MMSE. WHO (27) defines intrinsic capacity as the combination of an individual’s physical and mental abilities. In the Integrated Care for Older People (ICOPE) guidelines, functional ability was defined as the combination and interaction of intrinsic capacity with the environment in which an older adult life. Thus, the evaluation of intrinsic capacity allows to identify conditions associated with its decline, providing an alternative to intervene in halting, decreasing, or reversing it, which, in turn, improves health and QoL of individuals aged 80 and over.

Some studies (28–31), mainly cohort studies, with oldest-old people and centenarians and using the geriatric nutritional risk index (GNRI), have associated nutritional risk with various aspects of health, not just the classic ones, observed in the present study, such as cognition, nutrient status (e.g., selenium), sexual hormones and bone blurriness, in addition to abdominal obesity.

Frailty is recognized as an important predictor of adverse health events (32–34), as demonstrated in the systematic review and meta-analysis conducted by Crocker et al. (35) which observed an association between frailty and QoL in all domains studied. Divergent results were noted in the present study, which showed no association between nutritional risk and QoL with cluster analysis. A possible explanation could be that the present study included community-dwelling individuals aged 80 and over who regularly attended reference centers, with medical care and socializing spaces for the older adults’ population, which may not represent the health and social conditions of most individuals aged 80 and over.

It’s important to mention that to our knowledge there are few studies with individuals over 80 years old from São Paulo, which impedes a better comparison with the results of the present study. However, when we look at recent data from the demographic Census (36), Brazil has about 208 million habitants, and it is estimated that 15% comprise the older population (about 31 million) and 1.8% (200.000) are over 80 years old in São Paulo.

Therefore, individuals aged 80 and over included in the present study partially represent the Brazilian older adults, as described by Mirandola and Morsch (37). Based on the National Health Survey (NHS) performed by Brazilian Institute of Geography and Statistics (BIGS), enrollment 1,498 aged 80 and over people from all over the country, in relation of ethno-racial features: 55.7% self-declared to be white, 42.7% black or brown, 1.1% yellow and 0.4% self-reported as indigenous (37). The higher proportion of white ethno-racial features among the participants included in the present study could be explained by locations where data was collected.

In addition, the NHS-BIGS study showed that 34.7% of Brazilians individuals aged 80 and over are unable to read and write, and among the remaining (65.3%): 20% were literate in courses for adults, 35.3% presented elementary education attended on regular or supplemental, 5% attended high school and another 5% presented an university degree. It’s important to note that Brazil is a very large country with many socio-economic and cultural differences among regions, and in the present study the southeast individuals aged 80 and over self-reported 6 years of schooling (median), while in the study conducted by Amorim (38) with 135 individuals aged 80 and over from the Teresina, in the state of Piauí (northeast region) found that 62.1% of the participants were illiterate. In this way, the high proportion of Brazilians individuals aged 80 and over who only have an elementary education is a reflection of the social organization of the beginning of the last century, when education was not a priority and younger people had to choose between work or study in order to contribute to the family income (37, 39).

In the present study, it was also detected an association between nutritional risk and unmarried status in individuals aged 80 and over. A few studies associated nutritional risk and marital status, but other studies observed some association with weight loss and sarcopenia. Alexandre et al. (40) described an association between absence of marital life and sarcopenia to older adults who participated of the Health, Well-being, and Aging study in 2010 (SABE study), while Góes et al. (41) related that unmarried men lose weight at a higher rate, showing a higher risk of sarcopenia for older adults living in Southern Brazil (EpiFloripa Study).

Household income was another predictor of nutritional risk, corroborating with other studies found in the scientific literature. Rossi et al. (42) states that insufficient income gets in the way of obtaining enough food to meet physiological and psychological needs, which consequently may promote food insecurity, highlighting the importance of identifying those at risk, in order to demand greater efforts to implement public policies to reduce social inequities, and thus contribute for the prevention, maintenance, and promotion healthy aging (43, 44). Based on data from the NHS-BIGS, the monthly income of Brazilian individuals aged 80 and over was R$ 2087.00 (USD 417.40) ± R$ 4358.73 (USD 871.75) (37), in accordance as observed in the present study. In general, the low income of Brazilians individuals aged 80 and over has been described in some studies (45, 46), however, the discrepancies observed in the incomes could be explained by the region in which the studies were conducted. The studies that described long-lived people with higher income levels (2 to 3 minimum-wages) were carried out in the municipality of Marilia, a municipality in the state of São Paulo (47), in the south of state of Minas Gerais (48) and the Brazilian Federal District (49), while the studies that observed an average income of 1 minimum wage were conducted in the city of Recife, state of Pernambuco (50). The participants of the present study live in São Paulo, one of the richest states in the federation and has better health services that can contribute to better living conditions when compared to other regions of the country. In addition, the increase in the older adults population creates numerous challenges, influencing consumption, the transfer of benefits, taxes, pensions, jobs, family organization and the health sector. Almost all of the individuals aged 80 and over participants of this study declared that their income came from retirement and/or a pension (94.2%, data not shown), which highlights the importance of the federal government benefits for Brazilian individuals aged 80 and over.

Taken together, our findings are in accordance with other studies that show a low purchasing power is one of the characteristics of the older population in Brazil and social benefits are the main income source (46, 47, 51). This national scenario requires the state to formulate and implement public policies to assist this population, especially associated with social security. This issue deserves to be widely and carefully discussed since the growing social security deficit is a consequence of the model in which the contributions paid by active workers are intended to cover the costs of benefits for retirees and pensioners (52).

There was an association between nutritional risk and female sex in this study, a very common finding in studies with older adults (53–56). In a longitudinal study conducted with community-dwelling older adults in Spain (57) association was observed between nutritional risk and female sex, as well as with the physical and psychological domains of the WHOQOL-BREF.

The feminization of old age is a phenomenon highlighted by gerontological literature and there is a predominance of women in the Brazilian older old population, with some small variations in the proportions among regions (37, 38, 45, 46, 58–64). Ferreira (58) showed that 60,4% of individuals aged 80 and over including in Health, Wellbeing and Aging Study (SABE), under the coordination of the Pan American Health Organization, were women. The feminization of aging is not an exclusively Brazilian phenomenon, but is also observed elsewhere in the world and needs attention (37, 46, 65–68). Collerton et al. (67) studied individuals aged 80 and over in the United Kingdom and reported that 59.9% of the women. The same was observed in the Niklasson et al. (66) study with older adults in Sweden, with 79.4% of women. Several factors may be associated with this increased expectancy for women, such as regular medical care, observed mainly in women aged 80 and over (69). Porciúncula et al. (50) reported that older adult men are more exposed to acute lethal diseases, such as heart ischemia. The predominance of the female population among the individuals aged 80 and over has important repercussions on the demands for public policies, since various studies show that despite living longer, women are susceptible to developing functional disabilities and multiple health problems and live with them for longer (37, 70).

The population aging may be considered a successful episode for any society, but it can also be a challenge to ensure, to these individuals to gain years of life, but also quality to live these additional years (71).

Noteworthy no association with nutritional risk and number of teeth has been detected, all participants of the present study self-reported with edentulism or severe tooth loss (IQR 0–1, Table 1) and the use of dental prostheses. This data could be justified by the whole history of the Brazilian individual aged 80 and over and their access to health services, in which the traditional model exposed them to a mutilating practice that solved pain on a one-off basis (72). Unfortunately, studies that address the oral health condition of the Brazilian population are scarce, despite tooth loss harming the quality of life and health (73–75). According to the National Health Survey (PNS), 75% of Brazilian older adults self-reported the use of some type of dental prosthesis (76), and the prevalence of tooth loss among older adults remained high (77). Thus, the improvement in the oral health conditions of Brazilian older adults remains a challenge to public health, which needs to emphasize preventive measures to avoid total edentulism, as well as planning the prosthetic rehabilitation of tooth loss.

Although cluster analysis is a commonly useful method for characterization of data sets, mainly when there is no previous knowledge about the data correlation, this analysis has not detected any associations between clusters and nutritional risk. Thus, this method was used in the initial stage of the predictive model’s construction, allowing to rank QoL scores and sociodemographic characteristics in predicting nutritional risk. Random Forest was the method that presented the best performance for predicting nutritional risk (higher accuracy, sensitivity, and specificity). The comparison between RF and XBGoost model allowed us to verify the consistency among the variables which are most important to predict nutritional risk, and the performance of these ensemble methods. Several set models have been tested by both algorithms to improve predictive ability, and they have been frequently used with good results in the healthcare field (78–80).

It was very complicated to compare the results of the present study with previous studies found in scientific literature, due to the scarcity of studies with a similar proposal that considered the use of machine learning methods to predict nutritional risk in individuals aged 80 and over. Despite this, Lee et al. (81) used five algorithms to identify the factors that affect the health-related quality of life of older adults with chronic diseases, including RF. The authors demonstrated that the stepwise logistic regression (SLR) model was the most accurate (0.93), and the most important predictors were income, diagnosis of chronic disease, depression, discomfort, and perceivedhealth status. Prati (82) investigated the variables correlates of QoL, such as happiness, and life satisfaction in European adults aged 50 and over, using machine learning techniques, and concluded that the most important categories were physical health and subjective life circumstances. In addition, the most important categories correlating with happiness were sociodemographic factors, country of residence, and psychological variables (82).

In the present study, physical condition (evaluated by WHOQOL-BREF and handgrip) and cognitive performance (evaluated by MMSE) were the main predictors of nutritional risk by RF analysis. However, when WHOQOL-OLD was used as a predictor, the variables of muscle strength, income, and cognition presented higher levels of influence. Alonso et al. (83) suggested that lower handgrip strength is correlated with muscle strength in the lower limbs, which could be used as a proxy indicator of overall strength for screening older adults, since muscle weakness decreases functional capacity, which, in turn, can lead to dependence. In a recent study, Peterson et al. (84) provided initial evidence of age acceleration in older people with lower handgrip strength and loss of strength over time. Taken together, these findings suggest that the maintenance of muscle strength positively influences healthy aging and QoL.

Cognition appeared as one of the main predictors in both models used in the present study. According to Kwan et al. (85), dietary pattern is a modifiable risk factor associated with cognitive frailty. Neuroinflammation and oxidative stress are some of the mechanisms that affect the cognitive capacity of older adults, and weight loss, reduced energy, and reduced nutrient intake are associated with changes in body composition, physical abilities, and increased risk of frailty.

The main clinical implications are related to the importance of conducting multidimensional studies in an attempt to identify the physical, psychological, and social conditions, as well as their connections. Health professionals must be attentive to detect the decline of physical and cognitive capacity, as well as nutritional risk of individuals aged 80 and over.

Nutritional risk is a modifiable condition that affects quality of life. Older adults with cognitive and physical frailty who show signs of malnutrition need to be monitored, since interventions should be implemented to prevent worsening of the situation, and even to reverse it altogether. Another important point regards results that reinforce the need to implement social programs for vulnerable families with older adults, contributing to their access to healthy foods. In addition, the findings show the fundamental importance of specific, interdisciplinary interventions to preserve the independence and autonomy of individuals aged 80 and over.

## 5 Limitations

It is important to mention that, although this is a multicenter study, data were collected in convenient locations, and it was only possible to obtain a small, non-probabilistic sample size. Therefore, there is a selection bias in the sample, since the participants had a specific sociodemographic (as ethno-racial features; schooling; number of teeth) profile and may not represent the general characteristics of the Brazilian population and is not representative of individuals aged 80 or over. In addition, individuals aged 80 and over can present sensory alterations, mainly cognitive, auditory, and visual, which could influence their ability to remember and self-report their health conditions and QoL. The exclusion of other factors in the tests of predictors set evaluated in the present study may have also influenced its outcomes. Finally, the findings do not imply causality due to the cross-sectional nature of the study.

## 6 Final considerations

Our findings demonstrate that for individuals aged 80 and over including in the present study, several factors can predict nutritional risk, such as lower muscle strength, worse cognitive ability, lower income, female sex, and unmarried status. However, there was no significant association between nutritional risk and quality of life (QoL) and cannot be representative for all individuals aged 80 or over. In addition, the study showed that the random forest (RF) analysis method presented better accuracy, sensitivity, and specificity when compared to other ensemble methods, for the sample of the study. This suggests that machine learning could be a useful tool for predicting nutritional risk for individuals aged 80 and over, but need to be confirm if it would be also useful for other individuals aged 80 or over. Overall, the study highlights the importance of assessing nutritional risk in individuals aged 80 and over and identifying the associated factors to contribute to development interdisciplinary interventions proposals.

## Data availability statement

The raw data supporting the conclusions of this article will be made available by the authors, without undue reservation.

## Ethics statement

The studies involving humans were approved by Research Ethics Committee of São Judas Tadeu University (CAAE 49987615.3.0000.5404 and approval number 09069419.8.0000.0089). The studies were conducted in accordance with the local legislation and institutional requirements. The participants provided their written informed consent to participate in this study.

## Author contributions

AA, MB, AM-L, and RA: conception and design of the research, conceptualization, project administration, methodology, resources, and supervision. GB, VS, AA, MB, and RA: design of the paper, writing, and reviewing of manuscript. VS, AA, and GB: interpretation and analysis of the data. GB, GM, and DS: acquisition of the data. All authors contributed to the article and approved the submitted version.
