# Supplementary material for: Quality of life and socio-demographic factors associated with nutritional risk in Brazilian community-dwelling individuals aged 80 and over: cluster analysis and ensemble methods
Source: Front Nutr. 2024 Jan 3;10:1183058. doi: 10.3389/fnut.2023.1183058 (PMC10792032; doi:10.3389/fnut.2023.1183058)
Supplement: Supplementary file 1 [file Data_Sheet_1.PDF]

Supplementary Material

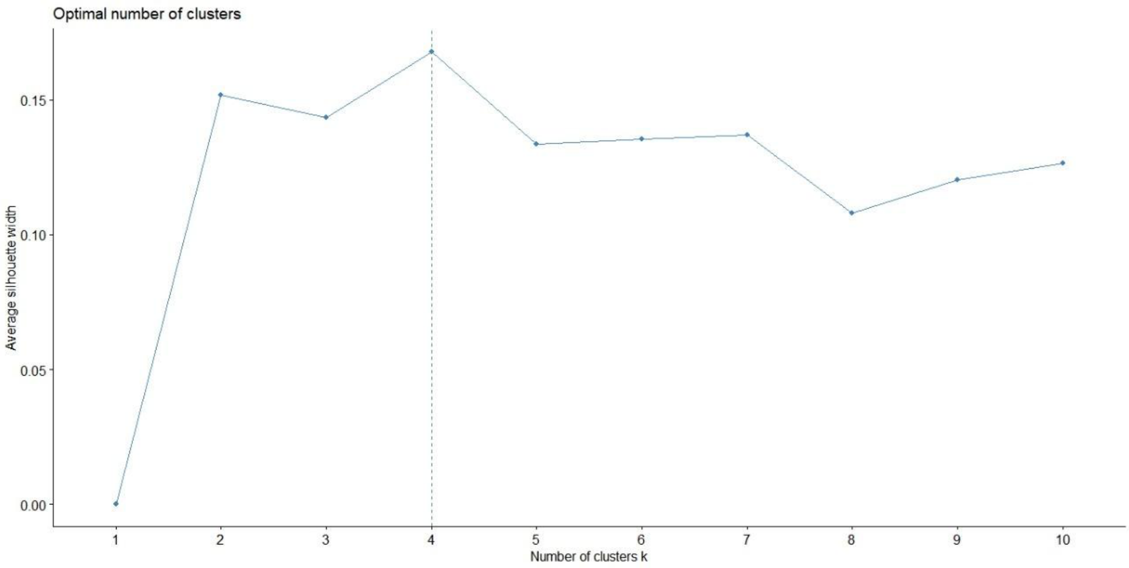

Figure S1. Optimal number of clusters.

Table S1. Key parameters of the Random Forest and XGBoost models.

Algorithm	Hyparameter	Value	
Random Forest	ntree	300	*Number of trees to grow.*
	mtry	3	*Number of variables randomly sampled as candidates at each split.*
	nodesize	5	*Minimum size of terminal nodes.*
	importance	True	*Should importance of predictors be assessed?*
	keep.forest	True	*If set to FALSE, the forest will not be retained in the output object.*
	seed	123	*For reproducibility.*
XGBoost	nround	300	*Max number of boosting iterations.*
	max_depth	2	*Maximum depth of a tree.*
	lambda	2	*Regularization term on weights.*
	nthread	2	*Number of threads.*
	eval_metric	auc	*Evaluation metrics for validation data.*
	eta	0.05	*Control the learning rate.*
	objective	binary:logistic	*Specify the learning task; Logistic regression for binary classification. Output probability.*
	print_every_n	100	*Print each n-th iteration evaluation messages.*
	seed	123	*For reproducibility.*

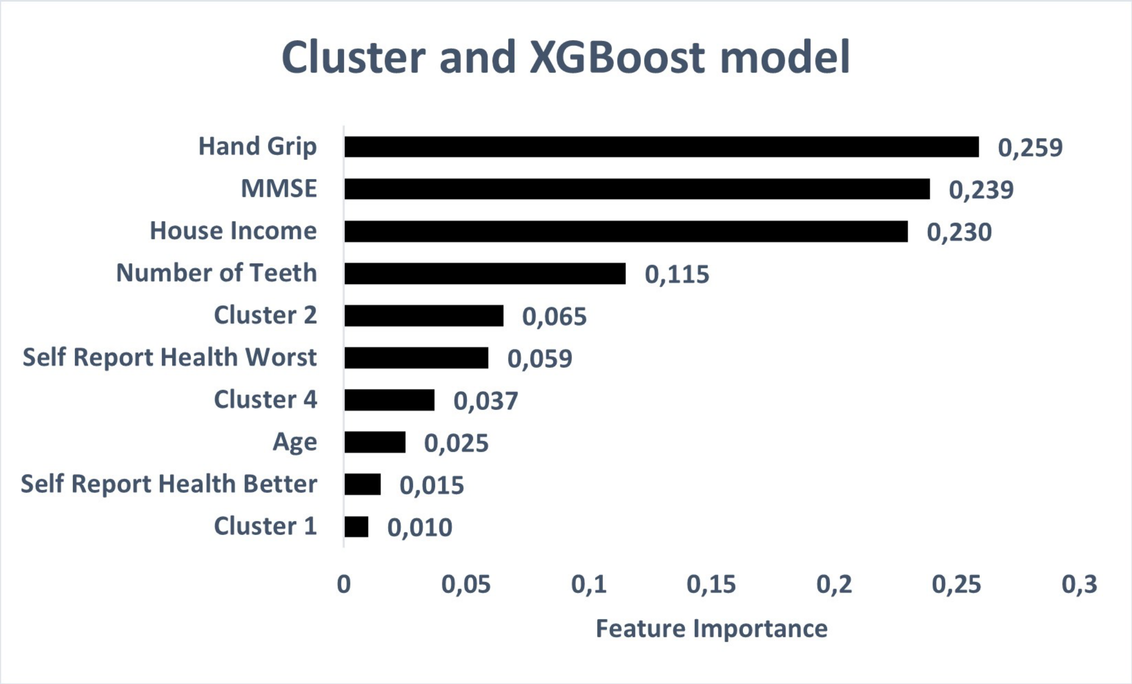

Figure S2. Predictors of nutritional risk with cluster quality of life and sociodemographic characteristics, self-reported-health and number of teeth, Mini-Mental State Examination (MMSE), and handgrip to individuals aged 80 and over analysed by XGBoost model.

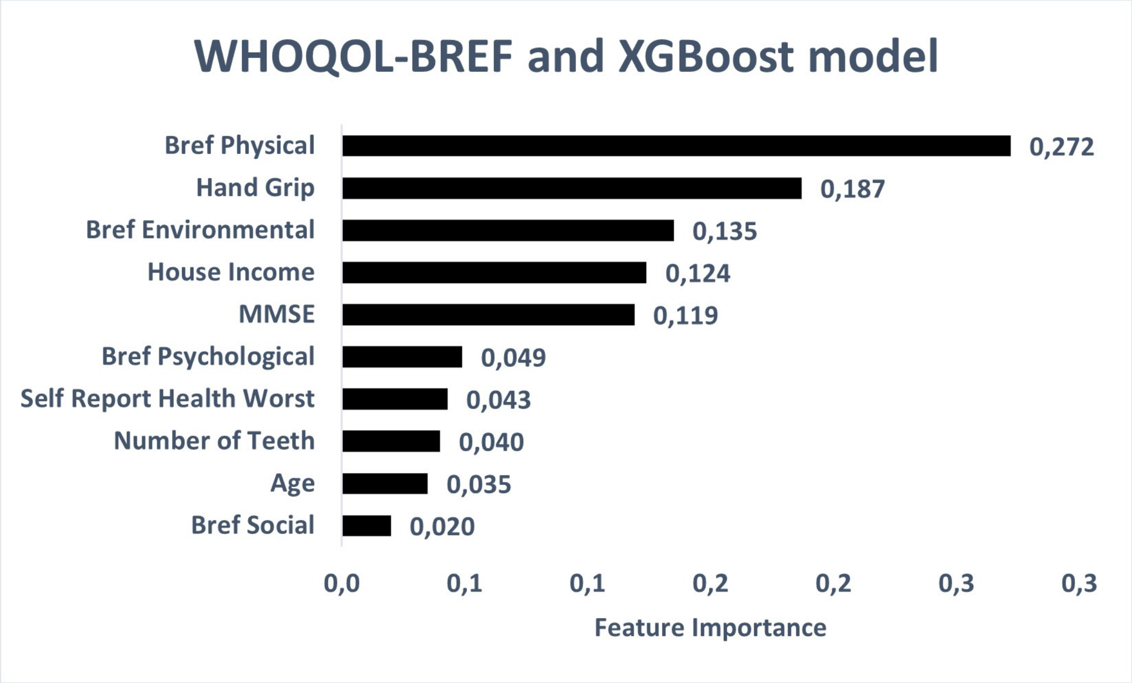

Figure S3. Predictors of nutritional risk with scores of WHOQOL-BREF and sociodemographic characteristics, self-reported-health and number of teeth, Mini-Mental State Examination (MMSE), and handgrip to individuals aged 80 and over analysed by XGBoost model.

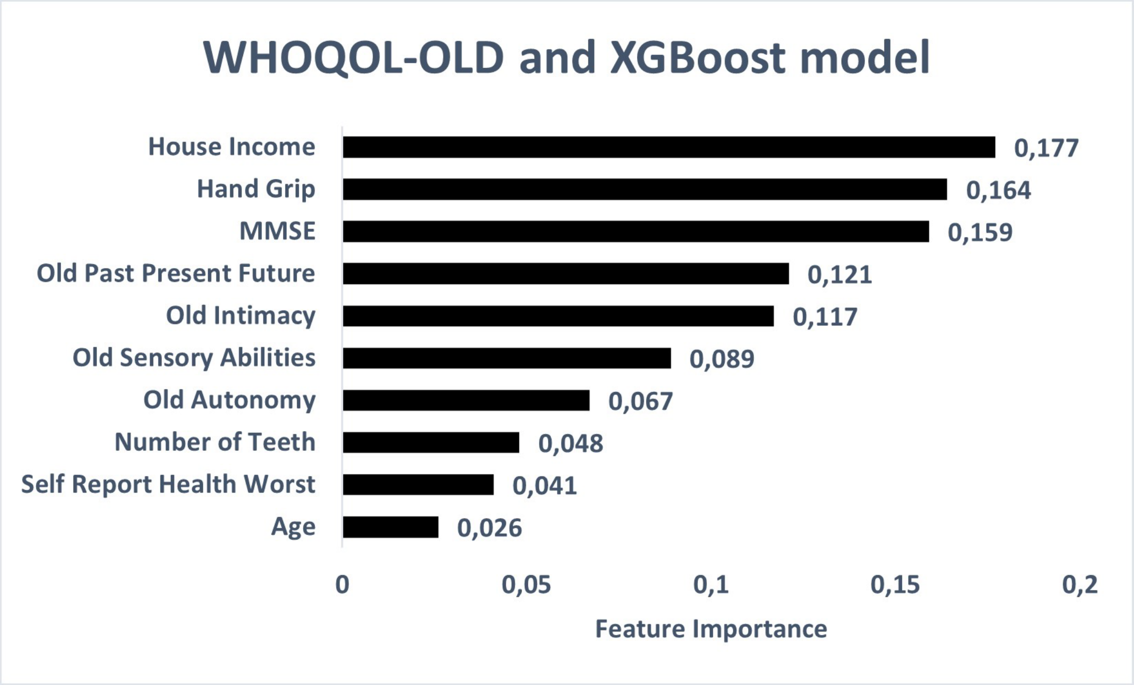

Figure S4. Predictors of nutritional risk with scores of WHOQOL-OLD and sociodemographic characteristics, self-reported-health and number of teeth, Mini-Mental State Examination (MMSE), and handgrip to individuals aged 80 and over analysed by XGBoost model.
